# *P. falciparum* Invasion and Erythrocyte Aging

**DOI:** 10.3390/cells13040334

**Published:** 2024-02-12

**Authors:** María Fernanda Alves-Rosa, Nicole M. Tayler, Doriana Dorta, Lorena M. Coronado, Carmenza Spadafora

**Affiliations:** Center of Cellular and Molecular Biology of Diseases, Instituto de Investigaciones Científicas y Servicio de Alta Tecnología (INDICASAT AIP), City of Knowledge, Panama City 0843-01103, Panama; mariaalves@indicasat.org.pa (M.F.A.-R.); ntayler@pa.estee.com (N.M.T.); doriana@indicasat.org.pa (D.D.); lcoronado@indicasat.org.pa (L.M.C.)

**Keywords:** *Plasmodium*, erythrocyte, senescence, deformability, invasion, receptors, cytoadherence

## Abstract

*Plasmodium* parasites need to find red blood cells (RBCs) that, on the one hand, expose receptors for the pathogen ligands and, on the other hand, maintain the right geometry to facilitate merozoite attachment and entry into the red blood cell. Both characteristics change with the maturation of erythrocytes. Some Plasmodia prefer younger vs. older erythrocytes. How does the life evolution of the RBC affect the invasion of the parasite? What happens when the RBC ages? In this review, we present what is known up until now.

## 1. Introduction: The Malaria Burden

After more than 120 years of research, malaria is still on the list of the deadliest tropical diseases. Most commonly found in tropical and subtropical countries, the African region bears the highest burden of malaria cases and fatalities, which are primarily attributable to the *Plasmodium falciparum* species, but every year nearly half of the world population is at risk of contracting malaria. In 2021, there were an estimated 247 million cases of malaria worldwide and the estimated number of malaria deaths stood at 619,000 [1].

As if these numbers were not daunting enough, the increase in the average temperature and precipitation patterns is leading to an increase in the vulnerability to the disease, turning vectors with historically limited range of transmission into high-reaching ones, rendering populations more vulnerable due to their reduced acquired immunity [2].

The use of drugs creates pressure on the parasites, forcing them to be selected for the presence of resistance genes, for example, the *pfcrt* gene, which is known to confer resistance against chloroquine [3,4,5], and the *pfmdr1* gene [6,7], which enable the parasite to expel multiple drugs. A study from the last decade reported that approximately 2 million known drugs were screened for antimalarial activity, of which more than 13,000 were found to be effective against the intraerythrocytic stage of the parasite [8].

## 2. Malarial Erythrocyte Receptors and *Plasmodium* Ligands

The identification of crucial ligand–receptor interactions involved in parasite erythrocyte entry is a venue that has been explored to tackle the disease, as their blockage may lead to the development of multivalent and more effective vaccines [9,10].

In studies focused on determining the proteins involved in the invasion process of *P. falciparum*, it was made clear that the physical interaction of the merozoite (the invading stage) with its proper receptor in the red blood cell (RBC) is a well-orchestrated process and that the integrity of the RBC membrane with its scaffold of proteins is crucial for this interaction to be successful. Invasion mechanisms used by the merozoite involve several steps that have been well described throughout the years [11].

As far as it is known, the first merozoite–host interaction is reversible, but it becomes more stable as the merozoite starts expressing some proteins on its surface. Microneme proteins form strong linkages between the parasite and the erythrocyte, helping rhoptry proteins in the final entry of the merozoite into the host cell. These initial steps in *Plasmodium* invasion are followed by the deformation of the erythrocyte membrane to form a parasitophorous vacuole membrane, in which the merozoite encapsulates itself after the shedding of surface proteins into the extracellular environment [12,13].

Based on the host molecules used by the parasite and their response to enzymatic treatment, two main invasion pathways for *P. falciparum* have been defined. One is based on the interaction of the parasite with sialic acid (SA) residues and the other pathway works in a manner that is independent of these molecules [14].

The known receptors for the SA-dependent pathways are Glycophorin (Gly) A [15,16,17,18]. Glycophorins are heavily glycosylated transmembrane sialo glycoproteins, which partly explains why these multi-abundant proteins in the erythrocyte membrane are responsible for the SA-dependent invasion by *P. falciparum*. These molecules have been characterized as carrying the antigens for several human blood groups: Gly A and Gly B carry the MN and SS blood groups, and Gly C carries the Gerbich blood group system [19,20]. Given the abundance of Glys on the RBC cell surface, it is likely that they also serve as substrates for glycosylation, which provides the RBC with a negatively charged complex glycan “coat” that allows for their circulation without adherence to other cells or walls of blood vessels [21].

As expected, the SA-dependent pathway efficiency is reduced upon enzymatic treatment of the erythrocyte with neuraminidase and the subsequent removal of sialic acid residues [22]. For this pathway, there is a redundancy in the parasite, comprising mainly several ligands of *P. falciparum* belonging to the Erythrocyte Binding Ligand (EBL) proteins and Erythrocyte Binding Antigens (EBAs).

Four main *P. falciparum* ligands have been identified for the SA-independent invasion: erythrocyte-binding antigen-175 (EBA-175), erythrocyte-binding antigen-181 (EBA-181), erythrocyte-binding ligand-1 (EBL-1), and erythrocyte-binding antigen-140 (EBA-140) [23]. It is generally accepted that Gly A is the receptor for the EBA-175 ligand, but *P. falciparum* can also invade erythrocytes using Gly B through EBL-1 [24] and through *P. falciparum* EBA-140 using Gly C as a receptor [25]. Regarding these receptors, Dankwa et al. reported that GPA and GPB are the key ones involved in the *P. falciparum* invasion route into human erythrocytes [26], probably due in part to their abundance on the surface of the erythrocyte.

The SA-independent parasite invasion ligands are dominated by the reticulocyte binding homologs family (PfRBL or PfRh) [18,27]. The family of sialic acid-independent or neuraminidase-resistant receptors [28,29,30] includes Receptor Z [27], Complement Receptor 1 (CR1) [31,32], Basigin (BSG) [33,34,35], and CD55 [36].

Receptor Z is used by the *Plasmodium falciparum* W2-mef and 3D7 strains. In related studies, the *P. falciparum* reticulocyte binding protein homolog 2b (PfRH2b) bound to RBC via this putative receptor, which was resistant to trypsin and neuraminidase treatment but sensitive to chymotrypsin [11]. Another ligand proposed for receptor Z is the Erythrocyte binding antigen-181 (EBA-181), for which no receptor is known [37].

The Complement Receptor 1 (CR1), also known as CD35 (cluster of differentiation 35), is an important polymorphic glycoprotein on the membrane surface of erythrocytes and many other nucleated cells. It is highly sensitive to treatment with trypsin. Along with other proteins, CR1 is also a regulator of the complement system, where it helps the RBC to avoid autologous complement attack [38].

The activation of a complement on the RBC membrane may occur due to the deposition of naturally occurring IgG autoantibodies, leading to the accumulation of C3b/C4b on the cell surface. In this scenario, C3b molecules bound to CR1 are deactivated by Complement Factor I, preventing erythrocytes against complement or phagocytosis-mediated destruction [38,39].

This feature also takes place in *P. falciparum*-infected erythrocytes. In ring-stage infections, phagocytosis is almost entirely dependent on the intervention of CR1 complement activation. This role of CR1 is reduced, however, when more mature forms of the parasite become present.

CR1 levels have been associated with malarial susceptibility and/or severity of the disease in different population groups [40]. Spadafora et al. observed that the amount of these molecules per erythrocyte varies depending on the donor and that levels of CR1 decrease in older erythrocytes when compared with younger ones [31]. The *P. falciparum* protein reticulocyte homology 4 (PfRh4) was reported as the CR1 ligand on the parasite [41].

Basigin (BSG), also called extracellular matrix metalloproteinase inducer (EMMPRIN) or CD147, is a type I transmembrane protein of the immunoglobulin (Ig) superfamily. This molecule was shown to associate with monocarboxylate transporters (MCTs) or plasma membrane calcium ATPases (PMCAs) expressed on the RBC surface [42,43]. MCTs are involved in a proton-coupled exchange of lactate or pyruvate across plasma membranes. The actions of both MCTs and PMCAs have been associated with the development of severe malaria [44,45], especially the presence of polymorphisms in PMCA [46].

The PfRh5 protein was found to bind specifically to BSG [33] at two highly glycosylated extracellular Ig domains [42], both of which contact PfRh5 [47]. The specific blockade of the PfRH5 binding to Basigin, coupled with PMCA or Basigin-MCT1, inhibits the parasitic invasion process [35]. PfRh5 is the only essential invasion ligand within the PfRh family. Both laboratory-adapted and field strains of *P. falciparum* follow a Basigin-dependent invasion route, corroborating the critical nature of these receptor-ligand interactions in erythrocyte invasion [48].

**Figure 1:**
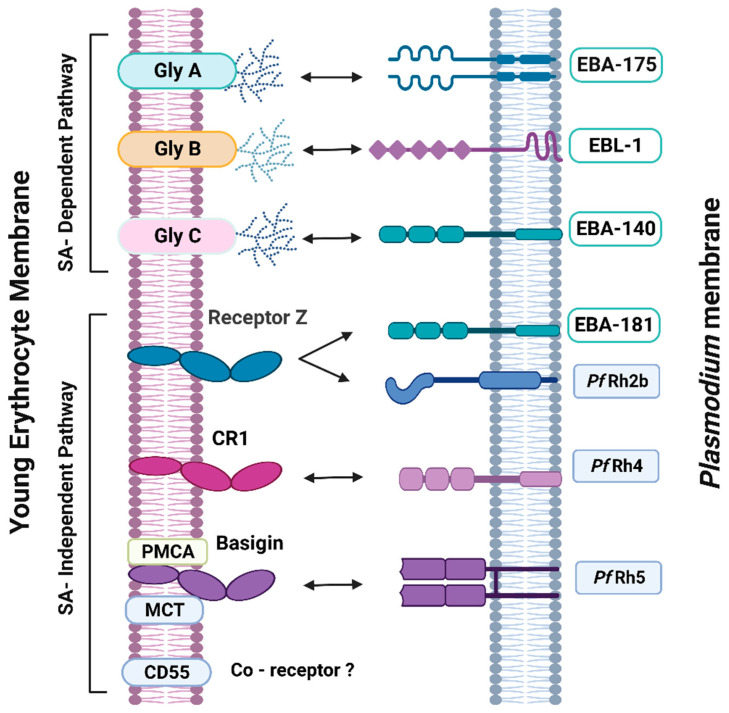
Main receptors and ligands involved in *P. falciparum* invasion of human erythrocytes. Gly A: glycophorin A. Gly B: glycophorin B. Gly C: Glycophorin C. CR1: Complement Receptor 1. PMCA: plasma membrane calcium ATPase. MCT: monocarboxylate transporter. CD55: Decay-Accelerating Factor (DAF). EBA-175: Erythrocyte Binding Antigen-175. EBL-1: Erythrocyte Binding Ligand-1. EBA-140: Erythrocyte Binding Antigen-140. EBA-181: Erythrocyte Binding Antigen-181. PfRh2b: *P. falciparum* Rhoptry homolog 2b. PfRh4: *P. falciparum* Rhoptry homolog 4. PfRh5: *P. falciparum* Rhoptry homolog 5. SA: Sialic Acid. The illustration is not at scale. This figure is created with BioRender.com.

Decay-Accelerating Factor (DAF), also known as CD55, is a GPI-linked complement-regulatory protein that protects cells from lysis by complement [49]. DAF is an essential host factor for *P. falciparum* invasion. CD55-null erythrocytes are refractory to invasion in vitro by isolates of *P. falciparum* due to the lack of parasite attachment to the erythrocyte surface [36]. In addition, CD55 is a receptor for bacterial and viral pathogens on epithelial cells [50]. Figure 1 displays the main ligand and receptor couples described here.

While it is known that there are other molecules involved in the parasite entry process into the RBC, the identity of some of their cellular receptors or ligands in the parasite is uncertain, and in some other cases, they belong to the same family of the ones depicted in Figure 1 [51,52,53,54,55].

All these data suggest that the more receptors available on the RBC surface, the more efficient the invasion process, and thus, the parasite virulence. As a result, any qualitative or quantitative change in the RBC cell surface could negatively modulate the parasite invasion efficiency.

## 3. Erythrocyte Age-Related Markers

The aging of RBCs is a natural process that occurs during their circulation in the vascular system. Normal human erythrocytes have an average life span of about 115 days in circulation, after which they are engulfed by macrophages [56,57].

The molecular mechanism by which macrophages discriminate between new and aged erythrocytes is a subject of study. There is, at least, a consensus that this process involves the appearance in the erythrocyte of different age-related markers, including changes in the composition of the cell membrane [58].

One of the most commonly accepted erythrocyte aging signals is the aggregation of the anion exchanger Band 3 [59]. These molecules cluster together on the membrane due to the association of hemichromes on the cytoplasmic domain of Band 3 [60]. This protein undergoes conformational dynamics localized in a region previously identified as an erythrocyte senescence epitope and may act as the “molecular clock” in erythrocyte senescence [61].

Naturally occurring antibodies that bind to clustered Band 3 were proposed as mediators of senescent RBC clearance [62,63]. The elimination of aged RBCs also involves phagocytosis by liver and spleen macrophages triggered by the binding of autologous IgGs to the aggregated Band 3 [64,65].

Band 3 plays an extremely important role as the organizational center of erythrocytes. This protein is linked to multiple complexes through adaptor proteins and constitutes a component of the cytoskeletal spectrin-based network [66]. It also serves as a binding site for several membrane-associated proteins [67], including *P. falciparum* glutamic acid–rich protein (PfGARP) [68] and Glycosyl-phosphatidylinositol-anchored micronemal antigen (GAMA) [69], both of which are erythrocyte-binding proteins known to be involved in malarial parasite invasion. PfGARPs are potential ligands that bind to the surface of intact RBCs with Band 3 as their receptor [68]. They are detected in the trophozoite and schizont stages of the parasite, but not in the ring stage.

Like other proteins on the surface of erythrocytes, CR1 levels also decrease as a result of senescence [70]. Ripoche et al. analyzed the loss of these molecules from erythrocytes by measuring the CR1 activity and found that older erythrocytes showed a decrease in activity relative to younger ones [71].

CR1 loss has also been tied to vesicular liberation from the membrane of erythrocytes. Observing the natural degradation of the erythrocytic plasma membrane, Pascual et al. proposed that intact CR1 molecules were released in phospholipid vesicles [72], which contributes to the loss of erythrocyte surface and volume. Nowadays, the discovery of ubiquitous extracellular vesicles and microvesicles, which are also shed by RBCs, can explain this observation. The release of MVs is considered a strategy of the erythrocyte to remodel, condense, and change the surface area to volume ratio, in the case of younger, bigger reticulocytes [73] and to dispose of some wasted proteins and contents in the case of adult erythrocytes [74]. Among the proteins liberated with MVs are membrane proteins, such as glycophorins [75]. However, when studying EVs released from *P. falciparum*-infected erythrocytes, Correa et al. observed that RBC CR1, in the same way as glycophorin A, is also grabbed from the RBC membrane into shed particles, confirming the report of Pascual et al. This finding suggests that this protein is regularly lost during MV biogenesis, together with glycophorins: a process that should deeply change the shape of the erythrocyte [76].

Trautsch C et al. reported lower levels of glycoproteins and acetylcholine in older RBC populations in vitro and in vivo [77]. Miyahara K et al. demonstrated an in vivo loss of glycoconjugate molecules, i.e., sialoglycoproteins, macroglycolipids, low-molecular-weight glycolipids, and Band 3 glycolipids on the surface of normal aging or diabetic human erythrocytes [78].

The presence of cell adhesion molecules (CAMs), such as CD44, CD47, BSG, Gly-A, and phosphatidylserine (PS), decreases upon erythrocyte aging [79,80].

Regarding CD44, this molecule exists in several isoforms, but RBCs only express the shortest one. Its role is not fully understood, aside from that of encoding the Indian blood group antigens [81]. When erythroid cell CD44 expression was abrogated by CRISPR/Cas9 genome editing, erythropoiesis and enucleation of orthochromatic erythroblasts were not affected [82]. However, CD44 was shown to play a role in *Plasmodium falciparum* invasion. The parasite requires CD44 as a co-receptor for both Gly-A ↔ EBA-175 and Gly-C ↔ EBA-140 interactions. Moreover, EBA-175 triggers the phosphorylation of cytoskeletal proteins through a CD44-dependent pathway [83].

The highly glycosylated RBC surface protein CD47 signals the regulatory protein alpha (SIRPα) on the macrophage. SIRPα signals for inhibition of both phagocytosis and inflammatory response. This interaction leads to erythrocytes phagocytosis suppression [84]. Physiologically, CD47 functions to prevent RBCs from undergoing phagocytosis [85].

During the aging of red blood cells, surface components, such as sialoglycoproteins, specifically sialic acids (SAs), undergo detachment [86,87]. Human erythrocyte SAs were found to contain 95% N-acetylneuraminic acid (NANA) [88]. The electrical charge that arises from NANA carboxyls and other ionizable chemicals is one of the major physicochemical factors that govern cell interactions. Negatively charged RBC membrane promotes intercellular electrostatic repulsion avoiding erythrocyte aggregation [89].

An enhancement of the phagocytosis of *P. falciparum*-infected red blood cells was observed when CD47-SIRPα engagement was disrupted using either anti-SIRPα antibodies or SIRPα-Fc fusion protein. These findings highlight the significance of CD47-SIRPα interactions in the innate regulation of malaria [90].

Basigin, which is expressed on erythrocytes, plays a critical role in recirculating mature erythrocytes from the spleen into the general circulation. Coste et al. showed that erythrocytes accumulate in the red pulp of spleens of anti-CD147-treated mice [91,92].

Another feature of RBC aging is changes in the intracellular Ca^2+^ concentration and translocation of PS from the inner to the outer membrane, leading to PS exposure. This is attributable to a decreased flippase activity in senescent erythrocytes [93] and the activation of scramblase [94].

As mentioned elsewhere, senescent erythrocytes show a shrinkage effect caused by the loss of cell membrane, which is manifested in the form of microvesicles shed into the surrounding media [95,96]. As observed both in vivo and in vitro, under various conditions, vesicles usually contain hemoglobin, lipids, band 3, glycophorins, actin, lipid raft proteins, caspases, Fas, and extracellular PS [97,98,99]. The proteins contained in microvesicles can be derived from plasma or small G proteins and they are rapidly removed from circulation, as signaled by the presence of PS and immunoglobulin G (IgG) on their surface [100,101]. Cell contents and membrane parts, carried outside by microparticles, result in a diminished ability of the erythrocyte to deform.

A reduction in deformability is a feature of an RBC’s physiological aging and it is associated with mostly uncharacterized metabolic and structural alterations [102]. To assess deformability, the first method used was simple observation under the microscope, but over time, different methods have been developed, which have increased in complexity and accuracy: micropipette aspiration [103,104], simple and microfluidic filtration [105,106], atomic force microscopy [107], erythrocyte shape recovery [108,109], optical tweezers [110,111], and laser diffractometry [112].

Using a range of the above techniques, a strong and meaningful intercorrelation was established between the deformability of RBCs and the presence of certain proteins [113].

Senescent cells, which are unable to squeeze through narrow splenic slits, are retained and cleared from the blood circulation, preventing microvessels from being clogged by aged, rigid RBCs. The biconcave shape that is natural to erythrocytes is an important contributor to maintaining adequate blood flow [114] because it contributes to this flexibility. Certainly, the retention of less deformable RBCs is a crucial factor that influences the pathogenesis of malaria [115]. Studies using optical tweezers revealed mechanical changes in *P. falciparum-infected* RBCs. In fact, an increase of up to 10-fold in the continuous force–displacement curves was detected for different intracellular developmental stages of the parasite [116].

## 4. Effect of *Plasmodium* spp. Invasion on Mechanical and Molecular Erythrocyte Properties

When erythrocytes are infected with *P. falciparum*, their natural aging process is accelerated and many of the RBC-aging features appear in infected RBCs, even though they are young [117]. The intra-erythrocytic phase of *Plasmodium* infection is initiated by erythrocyte invasion by merozoites, followed by the asexual replication cycle, which progresses through the ring, trophozoite, and schizont stages, until the new release of merozoites, a step which is associated with the clinical symptoms of malaria [118].

It has been well documented that erythrocytes that have been infected with the *Plasmodium* parasite undergo changes in their membrane composition, particularly in components such as phospholipids and cholesterol, and how these are organized [119,120]. In addition, *P. falciparum* also elicits the formation of hemichromes and the aggregation of Band 3 molecules for further opsonization and phagocytosis of the infected RBCs [121,122,123].

There is also an effect seen on the osmotic, antigenic, transportation, and deformation properties of *P. falciparum*-infected erythrocytes [114,124]. This is also true for uninfected red blood cells when malaria infection takes place. In fact, infected red blood cells (iRBCs) cause a bystander effect, wherein RBCs hosting the parasite provoke changes in the physical properties of the surrounding non-hosting RBCs [125,126]. These rigidified, uninfected red blood cells are mainly removed by splenic macrophages [127].

Changes in the membrane of the erythrocyte that are induced by malaria infection also affect their deformability. As previously explained, this property is crucial for their intrinsic ability to pass through capillaries and other narrow passages of the vascular system.

As the parasite grows inside the RBC, the latter becomes rounder and wrinkled. During asexual stages, the membrane of iRBCs presents knobs, or protrusions, elicited by parasitic proteins known as Knob Associated Histidine–Rich proteins [128,129,130]. Knobs act as a scaffold for the presentation of PfEMP1, which is a protein known as the main actor for the adhesion of the infected RBCs to the endothelium [131].

**Figure 2:**
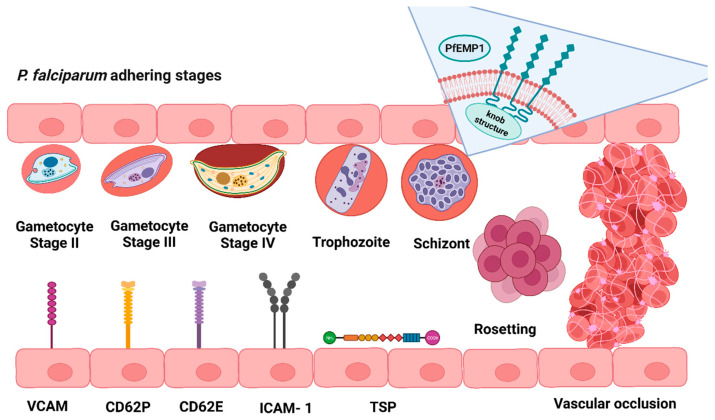
Cytoadherence events and main actors in the vascular system that takes place during *P. falciparum* infection and development in humans. ICAM-1: Adhesion Molecule-1, VCAM: Vascular cellular adhesion molecule, TSP: Thrombospondin, CD62P: P-selectin, CD62E: E-selectin, PfEMP1: *P. falciparum* Erythrocyte Membrane Protein 1. The illustrations are not at scale. The inset at the top of the figure represents a protein expressed in the membrane of each stage of *P. falciparum*: Gametocyte stages I–IV, trophozoite stage, and schizont stages. This figure is created with BioRender.com.

Red blood cells containing mature parasites exhibit unusual rigidity, and the primary factor responsible for their absence in the bloodstream is not their splenic uptake but an increase in their adherence to endothelial cells. The increased cytoadherence of the iRBCs to the endothelial lining of capillaries and deep tissues through Intercellular Adhesion Molecule-1 (ICAM-1), vascular cellular adhesion molecule (VCAM), Thrombospondin (TSP), P-selectin (CD36), E-selectin, and other molecules [132] is facilitated by specific parasite proteins exposed on the surface of the iRBCs (e.g., PfEMP1) [133]. Their increased adherence is responsible for the clinical occlusion of deep blood vessels in the brain when infected erythrocyte rosettes are formed, leading to the dreaded condition of cerebral malaria, which is a major complication in the malarial pathology [134,135] (Figure 2).

Most of the RBC deformability studies were performed on erythrocytes infected with the asexual forms of the parasites [136,137,138]. However, other lines of investigation on deformability were based on the effects that the sexual forms of the parasite exert on the erythrocyte [129,130]. It is worth noting that throughout their development, *P. falciparum* gametocytes alter the structural and mechanical characteristics of their host erythrocyte membrane.

The gametocyte has at least five (I–V) morphologically distinct stages in which it transforms; each one of them has different characteristics and effects on the host cell [139]. Through mathematical modeling, 3D imaging, and the use of transgenic parasites, Aingaran et al. demonstrated that early gametocytes increase the rigidity of the erythrocyte stages I to IV [129] and that immature sexual stages are enriched in proximity to erythroblastic islands [140]. Thus, the increased stiffness of immature gametocyte-infected erythrocytes could play a role in their entrapment within the bone marrow due to mechanical retention, favoring their maturation in the hematopoietic system. Mature gametocytes (stage V) exit this microenvironment possibly due to the restoration of their deformability [130], regaining their capacity to pass through narrow openings and be released into the bloodstream. This strategy can enable them to be available for ingestion by mosquitoes only once they have matured [131,141,142].

Many studies provided evidence about the role of several proteins expressed during the sexual stages of the parasite, such as Knob-associated histidine-rich protein, PfEMP3 [143], and *P. falciparum* Ring infected Erythrocyte Surface Antigen (RESA) [144] in the reduction of the erythrocyte deformability [131] (see a detailed list in Neveu et al., 2019). Furthermore, the expression of another family of proteins called STEVOR (Subtelomeric Variable Open Reading Frame) was studied in both the sexual and asexual stages of the parasite regarding this issue [145,146,147,148].

Varied functions in different parasite life cycle stages have been reported for the STEVORs and the Repetitive Interspersed Family (RIFIN) of proteins, such as rosetting, alteration of iRBC rigidity, and immune evasion [149,150].

Experiments performed on trophozoite-infected erythrocytes showed that endogenous expression of the STEVOR genes provokes important deformability effects on the erythrocytes, with the likely participation of RESA proteins [144,150]. This family of proteins is also expressed during the early stages of gametocytogenesis. STEVOR proteins are exported to the erythrocyte membrane during immature gametocyte stages [148,151,152,153].

The significance of these proteins lies in their potential involvement in, or induction of, the sequestration of gametocytes as a result of stiffness. This hypothesis is supported by data that reflect a high expression of these proteins in field samples, where parasite transmission is highly necessary for survival. Oddly, the expression of STEVOR proteins seems to be undetectable in in vitro clones, possibly because the mosquito stage of gametocytes is not necessary [147,148,154].

## 5. Changes in *Plasmodium* Invasion Strategies during Erythrocyte Senescence

As stated before, as the erythrocyte ages, changes in its membrane become evident, making this cell susceptible to opsonization and eventual phagocytosis. At first sight, erythrocyte senescence could impair the ability of parasites to survive due to decreased receptor availability and loss of deformability.

Indeed, the loss of SA receptors would pose another obstacle for the malaria parasite to overcome since most strains of *P. falciparum* primarily invade through the SA-dependent pathway, using alternate ways of invasion only when these glycoproteins are not available due to mutations, blockage with antibodies, enzymatic cleavage, or natural loss due to aging. However, Huang et al. showed that the RBC charge density is affected by the loss of NANA (SA) during erythrocyte aging [155]. The loss of this negative charge favors a stronger adhesion of RBCs to other cells and tissues.

The parasite, thus, maximizes the above scenario, adding the concomitant help of a reduced negative charge in the RBC to the endothelium-adhering action of its exported proteins, which provides an opportunity to better stick to capillaries and other vessels and avoid the spleen clearance.

To add to the remarkable resilience of this parasite, *P. falciparum* can infect red blood cells at different stages of the erythrocyte, including earlier maturation stages [156,157]. This observation could be added to the many resources it has, which might explain its high virulence and set it apart from other *Plasmodium* spp. that invade red blood cells at specific differentiation stages, such as *P. knowlesi*, *P. vivax*, and *P. ovale*. The entry of the latter into the cell is almost restricted to the very youngest circulating class of RBCs [158,159]. *P. malariae*, in contrast, prefers to invade older erythrocytes [160]. Receptors expressed in younger or older erythrocytes, for which each species probably expresses more ligands, might help to determine their invasion preference.

Just as *P. falciparum* relies on the presence of erythrocyte receptors, *Plasmodium vivax* [161,162] and *Plasmodium knowlesi* [22] also depend on the presence of the Duffy antigen (Fy) in its different polymorphic expressions. Fy is an almost obligatory receptor for the invasion of these parasites into host reticulocytes, wherein it is expressed at higher levels than in older RBCs [163,164]. This could be one of the reasons why *P. vivax* and *P. knowlesi* show a marked preference for the invasion of reticulocytes rather than the invasion of the more mature erythrocytes. This is not the case for *P. falciparum*, which is sufficiently resilient to survive a myriad of challenges, including that of erythrocyte aging.

Regarding the molecular changes experienced by senescent erythrocytes, which involve the loss of parasite receptors, either through aggregation and subsequent loss of function (e.g., Band 3), vesicle release, or downregulation, the parasite exhibits enough flexibility to compensate for their disappearance by adopting alternative entry pathways. While the diversity of receptors is reduced due to the aging process, the molecular availability of ligands remains high enough to promote infection, thereby conferring the characteristic virulence of *P. falciparum*. The extensive range of molecular options that enable the parasite to invade red blood cells complicates the development of effective blocking vaccines.

In terms of mechanical changes, the loss of elasticity cannot, by itself, be considered a marker of erythrocyte maturity, for this loss is also observed in young red blood cells infected with *Plasmodium* or even uninfected red blood cells subjected to bystander effects during infection [128]. Under normal circumstances, erythrocytes that have lost their deformability are captured by splenic macrophages, leading to their rapid removal. However, during infection, the parasite evades this process by expressing surface proteins on erythrocytes, allowing them to bind to the molecules present on endothelial cells. This enables the parasite to remain in vascular niches of organs, such as the heart, bone marrow, gastric mucosa, and even the brain, until reaching maturity [165], reducing its circulation in the bloodstream.

Under these circumstances, senescent red blood cells, or young red blood cells subjected to bystander effects when there is infection with *Plasmodium*, or even infected red blood cells that have not reached enough stiffness and remain in circulation, may act as decoys to partially saturate the phagocytic capacity of splenic macrophages, favoring the progression of the infectious process.

Some questions linger regarding the strategies of *P. falciparum* and other Plasmodia to select their receptors: Do they depend solely on their availability? How much of this preference could be attributed to changes in the geometry of the RBC due to the aging process? Do their preferred invasion receptors change over the lifespan of the RBC? Could other factors influence their invasion receptor selection?

Butcher et al., using *P. knowlesi*, reported that the invasion of RBCs might be affected by structural changes on the erythrocyte surface, but at the same time, they also showed that these changes are intrinsic to age progression [166]. Studies performed in *P. gallinaceum*-infected White Leghorn chickens demonstrated that this parasite prefers to invade young erythrocytes [167] and that this may be because young erythrocytes have larger amounts of phospholipids, which are required by the parasite invasion [168].

It is also known that choline, which is a precursor of the phospholipid composition of cells, is avidly taken in by *P. knowlesi*-infected simian red blood cells. The permeability of simian erythrocytes to choline was found to be considerably increased after infection by the malaria parasite [169,170]. In addition, it was reported that young malaria-infected erythrocytes showed an increase in choline permeability [171].

The increased expression of the key *P. falciparum* receptor, namely, Basigin, on early reticulocytes [172,173,174] could augment *P. falciparum* merozoite binding and invasion into reticulocytes, although this effect has not been directly demonstrated [156].

McQueen and McKenzie hypothesized that the different age-related preferences of RBCs according to species could be a strategy of *Plasmodium* spp. in areas where co-infection with different species takes place to keep a low-density parasitemia in balance with the ongoing availability of young, adult, and senescent RBCs [159].

## 6. Conclusions

From the concerted interactions between the merozoite and the RBC surface, it is evident that changes in an erythrocyte’s physical and molecular composition are crucial for the success or failure of the parasite entry and the development of its infective cycle.

Given that the aging of the erythrocyte may influence the number and availability of the receptors and that the deformability of the erythrocyte is critical for the invasion and development of *Plasmodium* parasites, an analysis of the relationship between RBC aging and malaria is very relevant to knowledge regarding the malaria pathogen. Understanding these dynamic changes holds substantial importance in the development of therapies to control malaria and vaccines to prevent it, and provides further insight into the comprehension of the pathogenesis of this disease.

For *Plasmodium*, the aging of red blood cells is not a determinant issue for its survival; on the contrary, it executes its plan for the survival of the species in a way that humans have not been able to overcome yet.
